# Numerical simulations of viscoelastic particle migration in a microchannel with triangular cross‐section

**DOI:** 10.1002/elps.202100121

**Published:** 2021-06-13

**Authors:** Gaetano D'Avino

**Affiliations:** ^1^ Dipartimento di Ingegneria Chimica, dei Materiali e della Produzione Industriale Università degli Studi di Napoli Federico II Naples Italy

**Keywords:** Numerical simulations, Particle migration, Triangular channel, Viscoelasticity

## Abstract

The migration of a spherical particle immersed in a viscoelastic liquid flowing in a microchannel with a triangular cross‐section is investigated by direct numerical simulations under inertialess conditions. The viscoelastic fluid is modeled through two constitutive equations to investigate the effect of the second normal stress difference and the resulting secondary flows on the migration phenomenon. The results are presented in terms of trajectories followed by the particles released at different initial positions over the channel cross‐section in a wide range of Weissenberg numbers and confinement ratios.

Particles suspended in a fluid with a negligible second normal stress difference migrate toward the channel centerline or the closest wall, depending on their initial position. A much more complex dynamics is found for particles suspended in a fluid with a relevant second normal stress difference due to the appearance of secondary flows that compete with the migration phenomenon. Depending on the Weissenberg number and confinement ratio, additional equilibrium positions (points or closed orbits) may appear. In this case, the channel centerline becomes unstable and the particles are driven to the corners or “entrapped” in recirculation regions within the channel cross‐section. The inversion of the centerline stability can be exploited to design efficient size‐based separation devices.

**Abbreviations**
ALE: Arbitrary Lagrangian‐Eulerian
GSK: Giesekus
PAA: poly(acrylamide)
PEO: poly(ethylene oxide)
PTT: Phan‐Thien–Tanner; SUPG, streamline upwing Petrov‐Galerkin

## 1 Introduction

The manipulation of particle trajectories through fluid viscoelasticity is a well‐established technique in many microfluidic applications [1, 2, 3, 4]. It is well known that the elasticity of the liquid induces a motion of the suspended particles orthogonal to the main flow direction, referred as migration phenomenon. Several theoretical, experimental, and numerical studies have clarified the role of fluid rheology on the particle migration. The lateral motion is induced by an unbalance of normal stresses around the particle that pushes it toward some equilibrium positions over the channel cross‐section. Both migration direction and velocity depend on many factors such as flow rate, fluid rheological properties, particle shape, confinement ratio [1, 4]. A relevant parameter that strongly affects the migration phenomenon is the shape of the channel cross‐section. Indeed, the cross‐section geometry determines the normal stress distribution in the fluid and, in turn, the number and the location of the equilibrium positions where the particles migrate. The device geometries commonly employed in microfluidic applications are cylindrical, slit, and square‐shaped channels. In cylindrical and wide‐slit channels (i.e., channels with a rectangular cross‐section with two sides much larger than the other two), the normal stresses have a minimum at the channel centerline or centerplane, respectively. In these channels, indeed, fluid viscoelasticity drives the particles to the central streamline (3D focusing) or centerplane (2D focusing) [5, 6, 7, 8]. This focusing behavior is observed for elastic, constant‐viscosity fluids. Shear‐thinning fluid generates an inversion of the migration direction near the channel walls, leading to the appearance of a separatrix such that particles migrate toward the channel centerline/centerplane or the wall depending on their initial position [6, 7]. A more complex scenario is observed for channels with a square or rectangular cross‐section with comparable side lengths. In this case, the normal stress distribution shows a minimum at the channel centerline as well as at the corners of the cross‐section. Consequently, particles aligned at the centerline coexist with particles migrated to the channel corners. As for the cylindrical/wide‐slit channels, shear thinning enhances the fraction of particles at the corners [9, 10]. Inertial effects can be exploited to reduce the particles at the corners and promote 3D focusing [11, 12, 13, 14].

In noncircular channels, particle migration is also affected by secondary flows, i.e., components of the velocity field that are orthogonal to the main flow direction [15, 16]. The secondary flows are related to the fluid second normal stress difference and are generally two to three orders of magnitude lower than the main flow velocity. Although the magnitude of the secondary flow is rather small, it can become comparable with that of the migration velocity affecting, in turn, the particle dynamics. A detailed study on the competition between secondary flows and migration phenomenon has been studied in a square‐shaped microchannel by numerical simulations [17]. The suspending fluid is modeled by two constitutive equations with the same rheological features (elasticity and entity of shear thinning) except the second normal stress difference. Particles suspended in a fluid without second normal stress difference migrate to the centerline of the channel or the corners depending on the initial position. This behavior is qualitatively independent of the Weissenberg number (denoted by Wi and defined below) and the confinement ratio. On the contrary, for relatively low confinement ratios, an additional stable equilibrium position in each octant of the cross‐section appears when the particles are suspended in a fluid with a non‐zero second normal stress difference. Particles initially released near these new equilibrium positions are driven there following spiraling trajectories. A variation of the confinement ratio and the Weissenberg number influences the competition between secondary flows and migration dynamics. Indeed, as the confinement ratio affects the magnitude of the migration velocity whereas the secondary flows magnitude does not change, the additional equilibrium points only appear at relatively low confinement ratios. As the confinement ratio increases, the migration velocity increases as well, overcoming the effect of secondary flows. Regarding the effect of the Weissenberg number, the migration velocity is linearly related to Wi whereas the secondary flow velocity scales with $Wi^{4}$. Hence, for small Wi‐values, the migration velocity is predominant and, consequently, only attraction at the centerline and corners is observed [17]. The relevance of secondary flows in noncircular channels has been reported by experiments as well [18]. Particles suspended in a PEO aqueous solutions (elastic, shear‐thinning fluid with a negligible second normal stress difference) flowing in a 2:1 rectangular channel are focused along the centerline or the walls. In contrast, small particles in a poly(acrylamide) PAA aqueous solutions (elastic, shear‐thinning fluid with a non‐negligible second normal stress difference), after being injected along the centerline or the walls, defocus due to the presence of secondary flows. Larger particles in PAA behave like in poly(ethylene oxide) solution.

Recently, a growing interest in studying the migration of particles in unconventional microchannel geometries is observed. The advancement of microfabrication techniques, indeed, allows the production of channels with complex shapes such as triangular, rhombic, semielliptical channels. Inertial focusing of rigid particles has been studied in triangular [19, 20, 21, 22], semicircular [19], trapezoidal [23], and rhombic [24] microchannels to understand how the cross‐section geometry affects the number and the focusing positions. Triangular channels have been also considered to study the inertial focusing of droplets and the effect of droplet size and deformability on the focusing positions [25]. In the context of viscoelastic microfluidics, particle migration has been investigated in rhombic [24], semielliptical [26], and triangular [26] microchannels. In all these geometries, elastoinertial particle focusing on a single line is observed in a wide range of flow rates. Due to the top–bottom asymmetry in the semielliptical and triangular shapes, the focusing position is near the channel bottom, resulting in an improvement of the detection sensitivity of impedance cytometer with coplanar electrodes fabricated along this channel side [26].

Motivated by these results, in the present work we carry out a detailed numerical study on the migration of spherical particles suspended in a viscoelastic fluid in a channel with triangular cross‐section. We assume inertialess conditions to single out the effect of fluid viscoelasticity on the particle motion. To highlight the influence of secondary flows, two constitutive equations that differ from the presence of the second normal stress difference are considered. The migration dynamics is analyzed through the trajectories of the particles released at different positions on the channel cross‐section in a wide range of Weissenberg numbers and confinement ratios.

## 2 Mathematical model and numerical method

**Figure 1:**
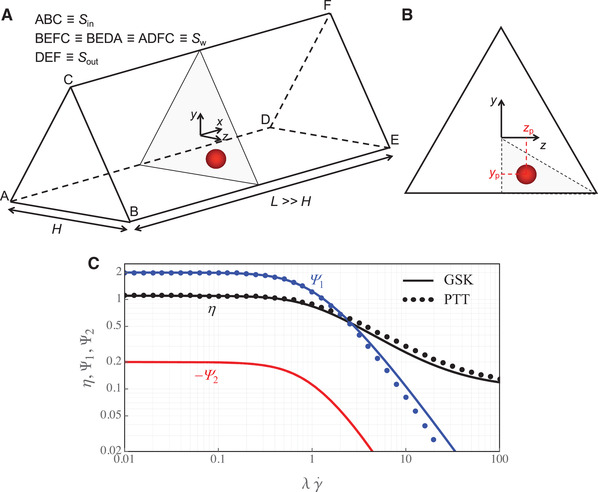
(A) Scheme of the problem investigated in this work. A microfluidic channel with an equilateral triangular cross‐section with side H and length L is considered. The origin of a Cartesian reference frame is set at the barycenter of the middle cross‐section shaded in gray. The inflow section is denoted by $S_{\text{in}}$, the outflow section by $S_{\text{out}}$, and the three side walls by $S_{w}$. (B) Channel cross‐section and the corresponding coordinates of the particle center. The simulations are carried out by considering the whole cross‐section. However, due to symmetry, particle dynamics is investigated only in the gray region corresponding to one‐sixth of the triangular cross‐section. (C) Rheological (dimensionless) properties of the GSK and PTT fluids in simple shear flow. The constitutive parameters are $\eta_{s}/\eta_{p}=0.1$ and α=ε=0.2.

The system considered in this work consists in a single, rigid, non‐Brownian, spherical particle suspended in a viscoelastic fluid flowing in a microchannel with an equilateral triangular cross‐section, as schematically depicted in Fig. 1. The particle diameter is $D_{p}=2R_{p}$ and the channel side is H. A Cartesian reference frame is selected with center coinciding with the barycenter of the middle channel cross‐section. The main flow direction is along the x‐axis, hence the cross‐section is parallel to the yz‐plane. The inflow, outflow, and wall boundaries are denoted by $S_{\mathrm{in}}$, $S_{\mathrm{out}}$, and $S_{w}$. A flow rate Q is imposed in inflow, no‐slip conditions are assumed at the channel walls and at the fluid–particle interface, and periodic conditions are applied between the inflow and outflow sections. We consider an infinitely long channel. Hence, the length L of the computational domain is selected sufficiently larger than the channel side to neglect the hydrodynamic interactions of the particle with its periodic images. The position of the particle center and the angular rotation are denoted by $x_{p}=\left(x_{p},y_{p},z_{p}\right)$ and $\Theta =(\Theta_{x},\Theta_{y},\Theta_{z})$. The particle motion is defined by the translational velocity $U_{p}=\left(U_{p},V_{p},W_{p}\right)$ and the angular velocity $\omega =(\omega_{x},\omega_{y},\omega_{z})$.

Assuming inertialess conditions for both fluid and particle, the fluid dynamics governing equations are the continuity and the momentum balance equations:

(1) ∇·u=0

(2) ∇·σ=0

(3) $$\sigma =-pI+2\eta_{s}D+\tau ,$$

where u, σ, p, I, $\eta_{s}$, and $D=(\nabla u+{\left(\nabla u\right)}^{T})/2$, τ are the fluid velocity, the total stress tensor, the pressure, the unity tensor, the viscosity of a Newtonian “solvent,” the rate‐of‐deformation tensor, and the viscoelastic stress tensor, respectively. The latter must be specified by selecting a constitutive equation. Following our previous work [17], we choose two constitutive equations, i.e., the Phan‐Thien–Tanner (PTT) model:

(4) $$\lambda \overset{\nabla }{\tau }+\exp\left[\frac{\varepsilon \lambda }{\eta_{p}}\text{tr}\left(\tau \right)\right]\tau =2\eta_{p}D$$

and the Giesekus (GSK) model:

(5) $$\lambda \overset{\nabla }{\tau }+\frac{\alpha \lambda }{\eta_{p}}\tau \cdot \tau +\tau =2\eta_{p}D.$$

In these equations, λ is the relaxation time of the fluid, $\eta_{p}$ is a viscosity, the symbol ${(}^{\nabla })$ denotes the upper‐convected time derivative:

(6) $$\overset{\nabla }{\tau }\equiv \frac{\partial \tau }{\partial t}+u\cdot \nabla \tau -{\left(\nabla u\right)}^{T}\cdot \tau -\tau \cdot \nabla u$$

and ε and α are (dimensionless) constitutive parameters. For ε=α=0, the two models recover the Oldroyd‐B constitutive equation. For positive values of these two parameters, both models predict (in shear) shear thinning for the viscosity and for the first normal stress difference coefficient $\Psi_{1}=N_{1}/{\dot{\gamma }}^{2}$ where $N_{1}$ is the first normal stress difference. In simple shear flow, the PTT model predicts a zero‐second normal stress difference $N_{2}$, whereas the GSK model predicts a negative value of this quantity. As mentioned in the previous section, the presence of a nonzero second normal stress difference gives rise to secondary flows in noncircular channels.

Regarding the boundary conditions, no‐slip is imposed at the channel walls $S_{w}$ and at the particle surface $S_{p}$:

(7) $$u=0\;\text{on}\;S_{w}$$

(8) $$u=U_{p}+\omega \times \left(x-x_{p}\right)\;\text{on}\;S_{p},$$

where x is the position vector of a point on the particle surface. As previously mentioned, periodicity is prescribed between the inflow and outflow sections, together with a flow rate in inflow:

(9) $${u|}_{S_{\text{in}}}{=u|}_{S_{\text{out}}}$$

(10) $$\left(\sigma \cdot i\right){{|}_{S_{\text{in}}}=\left(\sigma \cdot i\right)|}_{S_{\text{out}}}-\Delta p\;i$$

(11) $$\int_{S_{\text{in}}}u\cdot i\;dS=Q,$$

where i is the unit vector along the x‐direction and Δp is the pressure drop along the channel. The flow rate in Eq. (11) is imposed through a constraint where the associated Lagrange multiplier is identified as the unknown pressure difference Δp [27].

Finally, the hydrodynamic force and torque acting on the particle needs to be specified. Due to the inertialess assumption and of no “external” forces and torques, the particle is force‐ and torque‐free, that is, the total force F and torque T on the particle surface are zero:

(12) $$F=\int_{S_{p}}\sigma \cdot n\;dS=0$$

(13) $$T=\int_{S_{p}}\left(x-x_{p}\right)\times \left(\sigma \cdot n\right)\;dS=0$$

with n the outwardly directed unit normal vector on the particle surface $S_{p}$.

The inertialess assumption also implies that an initial condition for the velocity field is not needed. An initial condition for τ is, instead, required. We assume a stress‐free condition, that is, the stress is zero everywhere in the fluid at the initial time:

(14) $${\tau |}_{t=0}=0.$$

The solution of these equations gives the time evolution of u, p, τ, $U_{p}$, and ω. The last two quantities are used to update, at each time step, the particle position and rotation by integrating the following kinematic equations:

(15) $$\frac{dx_{p}}{dt}=U_{p}$$

(16) $$\frac{d\Theta }{dt}=\omega$$

with initial conditions $x_{p}{|}_{t=0}=x_{p,0}$ and ${\Theta |}_{t=0}=\Theta_{0}$. Note that Eq. (16) is decoupled from the other equations and can be left out being the orientation irrelevant for spherical particles.

The above equations can be made dimensionless using the hydraulic diameter $H_{\mathrm{hydr}}=\sqrt{3}H/3$ as characteristic length, the reciprocal of a typical shear rate $t_{f}=H_{\mathrm{hydr}}/\bar{U}=H^{3}/\left(4Q\right)$ as characteristic time with $\bar{U}$ the fluid average velocity, and $\eta_{p}/t_{f}$ as characteristic stress. Then, the Weissenberg number defined as:

(17) $$Wi=\frac{\lambda }{t_{f}}=\lambda \frac{8Q}{H^{3}}$$

appears in the equations. The Weissenberg number compares the fluid characteristic time λ and the flow characteristic time $t_{f}$. The Newtonian case corresponds to Wi=0. The other dimensionless parameters are the constitutive parameters α or ε, the viscosity ratio $\eta_{s}/\eta_{p}$, and confinement ratio:

(18) $$\beta =\frac{D_{p}}{H_{\mathrm{hydr}}}.$$

In this work, we fix the viscosity ratio to $\eta_{s}/\eta_{p}=0.1$ and α=ε=0.2. Figure 1C shows the viscosity η, the first and second normal stress difference coefficient $\Psi_{1}$ and $\Psi_{2}$ as a function of the dimensionless shear rate for both models in simple shear flow. With these constitutive parameters, the two models predict quantitatively similar trends of both the viscosity and the first normal stress difference coefficient. In what follows, all the quantities are dimensionless.

The set of equations has been solved by the finite element method. We implement the streamline upwind petrov‐galerkin (SUPG) formulation [28] together with a log representation for the conformation tensor [17, 29] to stabilize the numerical solution at high values of the Weissenberg number. The translational and the angular velocities of the particle are additional unknowns included in the weak form of momentum balance equation. Lagrange multipliers in every node of the particle surface are used to enforce the force‐ and the torque‐free conditions [30]. The arbitrary Lagrangian–Eulerian method is employed to manage the particle motion [31]. To reduce mesh distortion, the mesh is moved along the flow direction with a velocity given by the translational velocity of the particle along the same direction. The distortion of the mesh elements is, then, limited to directions lying over the channel cross‐section. Further details on the weak form, the solver, and the arbitrary Lagrangian–Eulerian implementation are reported elsewhere [17, 30].

Spatial and temporal convergence is verified for all the simulations presented in this work, especially those at the highest Weissenberg numbers. The mesh is made of tetrahedral elements and is refined near the particle where the largest gradients are expected. The number of mesh elements ranges between about 60 000 and 100 000, depending on the particle size, position, and the Weissenberg number. The length L of the domain is preliminarily checked to assure that the particle does not feel its image across the periodic boundaries. We found that a value of L=3H suffices to neglect periodic interactions for the largest confinement ratio (β=0.35) considered in this work. Finally, a time step size of Δt=λ/200 guarantees time convergent solutions for low and moderate Weissenberg numbers. At high Weissenberg numbers and for particles close to the channel boundary, we need to halve the time step to assure numerical stability.

## 3 Results and discussion

The particle migration phenomenon induced by fluid elasticity is studied through the analysis of the particle trajectories. The governing equations are solved in the whole geometrical domain. However, due to the symmetry, the particle is released only in one‐sixth of the triangular cross‐section indicated in grey in Fig. 1B. In the next sections, the results obtained for the PTT constitutive equation are first presented. Then, the dynamics of particles in a GSK suspending fluid is discussed.

### 3.1 Phan‐Thien–Tanner fluid

**Figure 2:**
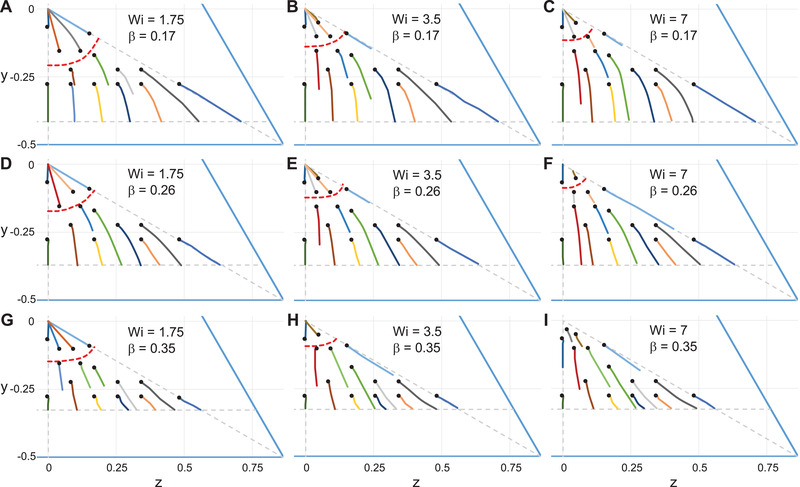
Trajectories of particles suspended in a Phan‐Thien–Tanner fluid projected on the channel cross‐section for different initial positions (denoted by black circles), Weissenberg number, and confinement ratio. The red dashed lines separate the trajectories toward the channel centerline from those toward the wall/corner. The horizontal dashed line delimits the region accessible to the particle whereas the vertical and diagonal lines delimit one‐sixth of the channel cross‐section where the particles are released.

The trajectories of a spherical particle released at different positions on the channel‐cross section and suspended in a PTT fluid are shown in Fig. 2. Several combinations of Weissenberg number and confinement ratio are considered. Specifically, the Weissenberg number increases from the left to the right (from Wi=1.75 to Wi=7) and the confinement ratio increases from top to bottom (from β=0.17 to β=0.35). The initial position of each trajectory is denoted by a black circle. The horizontal gray dashed line delimits the channel cross‐section such that the particle center cannot enter due to steric effects (i.e., the distance between the dashed line and the channel wall is the particle radius). The other gray dashed lines denote two heights of the cross‐section and delimit the region where the particles are initially positioned. Finally, note that the simulated time is not same for all the trajectories shown in this figure (and in the following figures). For instance, as discussed later, the simulations corresponding to particles migrating to the wall break down as the particle–wall distance becomes lower than a critical value. Hence, these trajectories are shorter than those corresponding to centerline migration. In general, we selected the initial position and the final integration time so that the resulting phase portrait gives a clear picture of the migration dynamics.

The trajectories reported in Fig. 2 show that migration can occur toward the channel centerline (i.e., the origin of the reference frame used) or toward the closest wall. The migration direction depends on the initial position and the same qualitative behavior is observed for all the Weissenberg numbers and confinement ratios investigated except the case at Wi=7 and β=0.35, where even the particles starting very close to the channel centerline migrate outwardly toward the channel wall (Fig. 2I). Except for the latter case, it is possible to identify a separatrix, denoted by a red dashed line, dividing the channel cross‐section in the two migration directions. This separatrix has been reported in the previous literature for other channel cross‐sections as well [6, 7] and is related to the shear thinning of the fluid.

**Figure 3:**
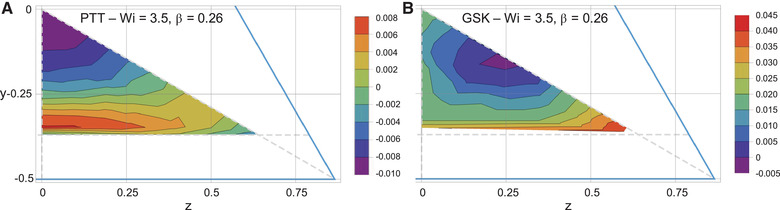
Contour plots of the magnitude of the migration velocity for a particle suspended in a Phan‐Thien–Tanner (A) and Giesekus (B) fluid for Wi=3.5 and β=0.26. The sign of the plotted velocity is taken opposite to the y‐component of the migration velocity. The horizontal dashed line delimits the region accessible to the particle whereas the vertical and diagonal lines delimit one‐sixth of the channel cross‐section where the particles are released.

In order to get information about the time needed for a particle to migrate, we reported in Fig. 3A the contour plot of the magnitude of the migration velocity (i.e., the velocity over the channel cross‐section) with sign opposite to the vertical velocity component, that is, $-\text{sign}\left(v\right)\sqrt{v^{2}+w^{2}}$. Hence, a negative or positive value denotes migration toward the centerline or the wall, respectively. The case Wi=3.5 and β=0.26, corresponding to Fig. 2E, is considered. It can be readily seen that the migration toward the wall is much faster than that toward the channel centerline. This is not surprising as particle migration is enhanced by confinement effects that are more relevant for small particle–wall distances. Note also that particles released near the separatrix migrate very slowly because they are near an unstable equilibrium point (a particle with center on the separatrix does not migrate at all). The maximum migration velocity is along the height of the triangle near the wall. As the particle further approaches the wall, the migration velocity decreases although it does not achieve a zero value when the particle (almost) touches the wall. To understand this behavior, let us consider the trajectories of the particles migrating toward the wall. They approximately lie on the extension of the straight line connecting the center of the cross‐section and the particle starting point. Hence, the particles rapidly accumulate along the lateral walls. Previous calculations, however, have demonstrated that, once the particles reach the walls, they start to roll along the wall [17]. Unfortunately, our simulations break down as soon as the particle–wall distance goes below a critical value due to the severe mesh distortion. However, by looking at the migration velocity components in the last steps of the simulations, we observe that the y‐component rapidly decreases whereas the z‐component increases. Hence, similarly to the migration in a square‐shaped channel [17], the particles, after quickly approaching the lateral walls of the channel, are expected to slowly roll along the wall toward the corners. Hence, the long‐time distribution of the particles suspended in a fluid with rheological features like those described by the PTT model in a triangular‐shaped channel is few particles aligned along the channel centerline and several particles at the corners.

Regarding the effect of the Weissenberg number and of the confinement ratio on the position of the separatrix, for a fixed value of β, the separatrix progressively moves toward the channel centerline as Wi increases. Hence, higher flow rates or larger relaxation times promote the migration of particles toward the corners. A similar effect is observed for increasing values of the confinement ratio. This is rather expected as, by increasing the confinement ratio, the steric effect increases as well, displacing the separatrix toward the centerline. To conclude this section, we mention that the bistable behavior reported in Fig. 2 is also found for lower values of the Weissenberg number and the confinement ratio.

### 3.2 Giesekus fluid

The results of migration of particles suspended in a GSK fluid are analyzed in this section. As previously remarked, the main difference between this constitutive equation and the PTT model is the existence of a nonzero second normal stress difference. The most relevant consequence in a noncylindrical channel is the appearance of components of the velocity field orthogonal to the main flow direction, referred as secondary flows. In a triangular channel, the secondary flows for Wi=3.5 are reported later in Fig. 5A. The secondary flows form six recirculation regions with sense (clockwise or counterclockwise) depending on the region of the cross‐section. In the one‐sixth of the cross‐section considered in this work the direction is counterclockwise. The secondary flows are two to three orders of magnitude smaller than the main flow velocity (see the color scale). Nevertheless, their presence may affect the migration dynamics as they can become comparable with the migration velocity [17].

**Figure 4:**
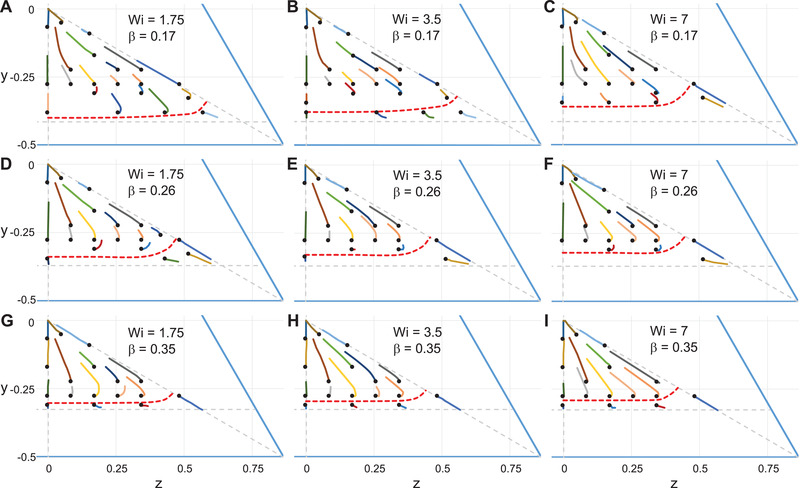
Trajectories of particles suspended in a Giesekus fluid projected on the channel cross‐section for different initial positions (denoted by black circles), Weissenberg number, and confinement ratio. The red dashed lines separate the trajectories toward the channel centerline from those toward the wall/corner. The horizontal dashed line delimits the region accessible to the particle whereas the vertical and diagonal lines delimit one‐sixth of the channel cross‐section where the particles are released.

**Figure 5:**
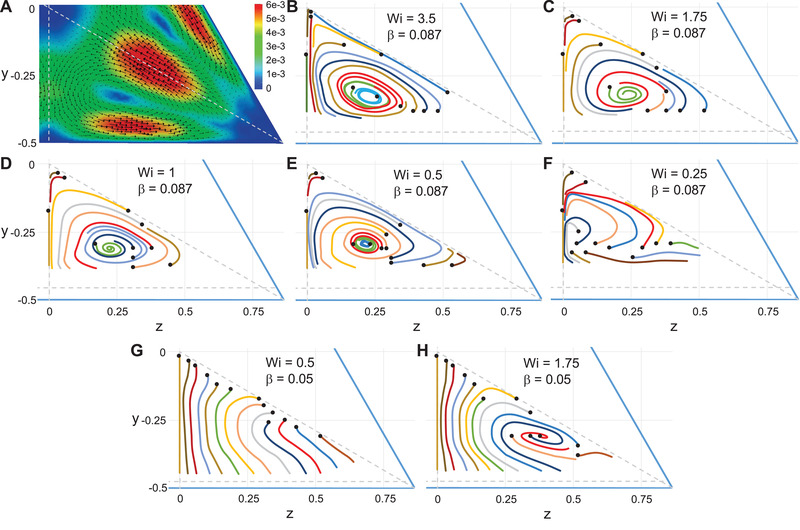
(A) Arrow plot of the secondary flows for a Giesekus fluid for Wi=3.5. The colors are the magnitude of the secondary flow velocity. (B–H) Trajectories of particles suspended in a Giesekus fluid projected on the channel cross‐section for different initial positions (denoted by black circles) and Weissenberg numbers. The confinement ratio is fixed at β=0.087 (B–F) and β=0.05 (G and H). The horizontal dashed line delimits the region accessible to the particle whereas the vertical and diagonal lines delimit one‐sixth of the channel cross‐section where the particles are released.

The trajectories of particles released at different initial positions are shown in Fig. 4 for the same combinations of Weissenberg number and confinement ratio previously considered. As for the PTT fluid, only the bottom‐right region of the channel cross‐section is analyzed. The scenario is remarkably different from the PTT case: for almost all the initial positions, the particles migrate toward the channel centerline. Migration toward the corner is only observed for those particles starting initially very close to the wall. This behavior is qualitatively independent of the Weissenberg number and the confinement ratio considered in these figures. The separatrices, denoted by dashed red lines, are located near the channel corner and the lateral wall. In some cases, we are unable to determine the trajectories of particles initially released very close to the wall as the simulations break down after few time steps. In these cases, the position of the separatrix is evaluated by extrapolating the migration velocity toward zero. In any event, the possible region characterized by wall attraction is so narrow to be negligible for any practical application.

Another difference with the previous constitutive equation is that the trajectories do not follow approximately straight lines. Rather, especially for the particles starting close to the bottom wall, the trajectories have an initial curvature in a counterclockwise sense up to becoming straight lines pointing to the channel centerline. Hence, the particles first slightly approach the opposite lateral wall of the channel cross‐section and then migrate toward the centerline. This effect is likely due to the secondary flows.

Regarding the migration velocity, we report in Fig. 3B the same quantity shown Fig. 3A for the PTT fluid. The case corresponding at Wi=3.5 and β=0.26 is considered. The maximum velocity toward the centerline is on the diagonal, almost at the midpoint between the channel centerline and the separatrix (corresponding to the zero‐velocity contour). A region near the corner characterized by a large migration velocity toward the corner is also visible. Due to the aforementioned numerical issues, the contour plot does not extend up to the dashed horizontal gray line (actually, the contours below the separatrix are obtained by interpolating the data of the few trajectories we were able to compute). Regarding the effect of the Weissenberg number and confinement ratio on the position of the separatrix, similar features of the PTT case can be observed, that is, the separatrix moves toward the centerline as the Weissenberg number and the confinement ratio are increased. A variation of the Weissenberg number on the separatrix position is less and less relevant as the confinement ratio is higher.

As previously reported for a square‐shaped microchannel [17], the effect of secondary flows becomes relevant as the confinement ratio is reduced. We, then, explored the particle trajectories by progressively decreasing the confinement ratio. Figure 5B reports the particle trajectories for Wi=3.5 and β=0.087. The scenario is remarkably different from the previous one. We observe that (i) the centerline becomes an unstable equilibrium point, (ii) the trajectories tend to a new attractor characterized by a limit cycle (see, e.g., the green, red, and cyan curves that reach the same closed periodic orbit), (iii) a new unstable equilibrium point appears within the limit cycle (inside the cyan orbit). We cannot assess whether the corner is still an attractor as our simulations break down for small particle–wall distances. Based on previous calculations [17] and on the initial transient dynamics of the present simulations before the occurrence of numerical issues, we expect that the corner is a stable equilibrium point and a separatrix similar to those reported in Fig. 4 exists. The qualitatively different phase portrait shown in Fig. 5B is due to the secondary flow velocities that become comparable with the migration velocity because of the small particle size.

We further explored the competition between secondary flows and migration phenomenon by reducing both the Weissenberg number and the confinement ratio. The results are summarized in Fig. 5C–H. Reducing the Weissenberg number from Wi=3.5 (Fig. 5B) to Wi=1.75 (Fig. 5C) while keeping the same confinement ratio β=0.087 leads to an inversion of the stability of the equilibrium point inside the limit cycle (Hopf bifurcation). Indeed, as visible from the green and red curves in Fig. 5C, the trajectories of particles initially released near such an equilibrium point converge there. Consequently, the limit cycle becomes unstable and the trajectories of particles outside the cycle follow spiraling orbits that move away from the cycle. The channel centerline is still unstable and the corner must be the attractor for these trajectories external to the limit cycle. As Wi is further decreased to Wi=1 and Wi=0.5 (Fig. 5D and E), the unstable limit cycle decreases in size and a smaller and smaller fraction of particles is attracted toward the stable equilibrium point (through spiraling orbits). Consequently, a larger number of particles is driven toward the corners. Figure 5F shows the phase portrait for an even smaller Wi‐value. Both the unstable limit cycle and the internal equilibrium point disappear and all the orbits converge to the corner.

By decreasing the confinement ratio to β=0.05, the phase portraits are qualitatively different as displayed in Fig. 5G and H. Comparing with the corresponding results at the same Weissenberg number in the same figure, the limit cycle and the internal equilibrium point are not present, and the only stable solution is the corner. Hence, regardless of the initial particle position, fluid viscoelasticity leads the particles to accumulate to the channel corners. This behavior is observed for both Wi=0.5 and Wi=1.75, the only difference being the orbits followed by the particles that are more similar to spirals in the latter case.

The analysis just presented clearly shows that the interplay between secondary flows and migration phenomenon is rather complex and gives rise to scenarios that are strongly dependent on the Weissenberg number and confinement ratio. As a general rule, the secondary flows weakly affect the particle transversal dynamics for sufficiently large particle sizes due to the high migration velocity induced by strongly confined systems. In contrast, for small particle dimensions, additional equilibrium solutions, such as stable positions or attractive orbits, appear.

A comparison of the particle migration dynamics between a triangular and a square cross‐section is in order. At sufficiently high Wi and β, the phase portraits shown in Fig. 4 are qualitatively similar to those reported in a channel with a square cross‐section under similar operating conditions. As β is reduced, in both channels, the existence of a stable limit cycle is observed for values of confinement ratios around 0.08–0.1 and Weissenberg numbers between 1.5 and 2. On the other hand, previous simulations in a square‐shaped channel showed that the intermediate equilibrium solutions disappear as Wi decreases to 0.5 [17], whereas this is not the case for a triangular cross‐section, as shown in Fig. 5E. A smaller value of the Weissenberg number is needed to get rid of the intermediate equilibrium positions in a triangular channel (Fig. 5F). This behavior can be justified by the different scaling of the secondary flows and migration velocities with Wi [17]. The behavior at smaller β shown in Fig. 5G and H is, instead, rather unexpected. As the particle size is reduced, the migration phenomenon induced by viscoelasticity should be less and less relevant and the secondary flows should rule the particle motion. Although the trajectories in Fig. 5G and H clearly reflect the effect of secondary flows, the particles (very slowly) are driven to the corner for any initial position. Simulations in a square‐shaped channel for β=0.05 are not available so we cannot assess whether this behavior is specific for the triangular cross‐section. The transition between β=0.087 to β=0.05 requires further investigation through other simulations at intermediate values of the confinement ratio.

Finally, when the confinement ratio is small enough so that secondary flows affect the particle motion, a relevant difference between the particle dynamics in square and triangular channels is that in the former geometry the centerline is characterized by a large basin of attraction (i.e., the most of the particles migrate at the centerline and a small fraction moves at the walls/corners), whereas in a triangular channel the migration is to the walls/corners regardless of the initial position. Hence, under these conditions, a channel with a square cross‐section has to be preferred for 3D particle focusing. Inertial effects are, then, needed to revert the stability of the centerline [26]. On the other hand, the inversion of the centerline stability due to a change of the confinement ratio observed in a triangular channel can be exploited to separate particles by size.

## 4 Concluding remarks

In this work, the migration of a rigid, spherical particle suspended in a viscoelastic fluid flowing in a microchannel with a triangular cross‐section is investigated by 3D finite element direct numerical simulations under inertialess conditions. Two constitutive equations, for example, the PTT and the GSK model, have been chosen in order to highlight the effect of the second normal stress difference and the resulting secondary flows on the particle dynamics. The migration phenomenon is studied through phase portraits over the channel cross‐section for Weissenberg numbers ranging from 0.25 to 7 and confinement ratios from 0.05 to 0.35.

Particles suspended in a fluid with no second normal stress difference migrate toward the channel centerline or the closest wall depending on the initial position. This behavior is observed for all the investigated combinations of Weissenberg numbers and confinement ratios. Once reached the walls, the particles are expected to (slowly) roll toward the closest corner. Hence, four equilibrium positions exists (the centerline and the three corners) corresponding to the regions of minimum shear rate. The curve that separates the two migration directions moves toward the centerline as Wi increases. For sufficiently high values of Wi and β the separatrix disappears and all the particles migrate toward the walls/corners.

A much more complex scenario is observed for particles suspended in a fluid with nonnegligible second normal stress difference due to the appearance of secondary flows. In this case, the results can be summarized as follows. For relatively large confinement ratios (β>0.15) migration prevails and the particles are mainly driven to the channel centerline; only a small fraction of particles moves to the closest corner. For smaller values of the confinement ratio, the secondary flows compete with the migration phenomenon giving rise to additional equilibrium solutions that coexist with the previous ones. Specifically, a stable limit cycle appears near the middle of the investigated region of the triangular cross‐section, and the channel centerline becomes unstable. Hence, depending on the initial position, the particles can be ‘entrapped’ in this vortex or migrate toward the corner. As Wi or β are reduced, the limit cycle decreases in size up to collapse with the inner equilibrium position. In this case, the particles are driven to the closest corner regardless of the initial position. The inversion of the centerline stability as the confinement ratio is varied can be exploited to design devices for separating particles of different size.

## Conflict of interest

The author has declared no conflict of interest.

## Data Availability

The data that support the findings of this study are available from the corresponding author upon reasonable request.

## References

[1] D'Avino, G. , Greco, F. , Maffettone, P. L. , Annu. Rev. Fluid Mech. 2017, 49, 341–360.

[2] Yuan, D. , Zhao, Q. , Yan, S. , Tang, S.‐Y. , Alici, G. , Zhang, J. , Li, W. , Lab Chip 2018, 18, 551–567. 29340388 10.1039/c7lc01076a

[3] Manshadi, M. K. D. , Mohammadi, M. , Monfared, L. K. , Sanati‐Nezhad, A. , Biotechnol. Bioeng. 2019, 117, 580–592. 31654394 10.1002/bit.27211

[4] Zhou, J. , Papautsky, I. , Microsyst. Nanoeng. 2020, 6, 113. 34567720 10.1038/s41378-020-00218-x PMC8433399

[5] Leshansky, A. M. , Bransky, A. , Korin, N. , Dinnar, U. , Phys. Rev. Lett. 2007, 98, 234501. 17677908 10.1103/PhysRevLett.98.234501

[6] Villone, M. M. , D'Avino, G. , Hulsen, M. A. , Greco, F. , Maffettone, P. L. , J. Non‐Newtonian Fluid Mech. 2011, 166, 1396–1405.

[7] D'Avino, G. , Romeo, G. , Villone, M. M. , Greco, F. , Netti, P. A. , Maffettone, P. L. , Lab Chip 2012, 12, 1638–1645. 22426743 10.1039/c2lc21154h

[8] Seo, K. W. , Jun Byeon, H. , Huh, H. K. , Lee, S. J. , RSC Adv. 2014, 4, 3512–3520.

[9] Del Giudice, F. , Romeo, G. , D'Avino, G. , Greco, F. , Netti, P. A. , Maffettone, P. L. , Lab Chip 2013, 13, 4263–4271. 24056525 10.1039/c3lc50679g

[10] Del Giudice, F. , D'Avino, G. , Greco, F. , Netti, P. A. , Maffettone, P. L. , Microfluid. Nanofluid. 2015, 19, 95–104.

[11] Yang, S. , Kim, J. Y. , Lee, S. J. , Lee, S. S. , Kim, J. M. , Lab Chip 2011, 11, 266–273. 20976348 10.1039/c0lc00102c

[12] Seo, K. W. , Kang, Y. J. , Lee, S. J. , Phys. Fluids 2014, 26, 063301.

[13] Wang, P. , Yu, Z. , Lin, J. , J. Non‐Newtonian Fluid Mech. 2018, 262, 142–148.

[14] Yu, Z. , Wang, P. , Lin, J. , Hu, H. H. , J. Fluid Mech. 2019, 868, 316–340.

[15] Xue, S. C. , Phan‐Thien, N. , Tanner, R. I. , J. Non‐Newtonian Fluid Mech. 1995, 59, 191–213.

[16] Debbaut, B. , Avalosse, T. , Dooley, J. , Hughes, K. , J. Non‐Newtonian Fluid Mech. 1997, 69, 255–271.

[17] Villone, M. M. , D'Avino, G. , Hulsen, M. A. , Greco, F. , Maffettone, P. L. , J. Non‐Newtonian Fluid Mech. 2013, 195, 1–8.

[18] Lim, H. , Nam, J. , Shin, S. , Microfluid. Nanofluid. 2014, 17, 683.

[19] Kim, J.‐A. , Lee, J. , Wu, C. , Nam, S. , Di Carlo, D. , Lee, W. , Lab Chip 2016, 16, 992–1001. 26853995 10.1039/c5lc01100k

[20] Kim, J.‐A. , Lee, J.‐R. , Je, T.‐J. , Jeon, E.‐C. , Lee, W. , Anal. Chem. 2018, 90, 1827–1835. 29271639 10.1021/acs.analchem.7b03851

[21] Mukherjee, P. , Wang, X. , Zhou, J. , Papautsky, I. , Lab Chip 2019, 19, 147–157. 10.1039/c8lc00973b 30488049

[22] Kim, J.‐A. , Kommajosula, A. , Choi, Y.‐H. , Lee, J.‐R. , Jeon, E.‐C. , Ganapathysubramanian, B. , Lee, W. , Biomicrofluidics 2020, 14, 024105. 32231759 10.1063/1.5133640 PMC7093208

[23] Moloudi, R. , Oh, S. , Yang, C. , Warkiani, M. E. , Naing, M. W. , Microfluid. Nanofluid. 2018, 22, 33.

[24] Kwon, J.‐Y. , Kim, T. , Kim, J. , Cho, Y. , Micromachines 2020, 11, 998.

[25] Choi, Y.‐H. , Kim, J.‐A. , Lee, W. , Micromachines 2020, 11, 839. 10.3390/mi11090839 PMC7570260 32906834

[26] Tang, W. , Fan, N. , Yang, J. , Li, Z. , Zhu, L. , Jiang, D. , Shi, J. , Xiang, N. , Microfluid. Nanofluid. 2019, 23, 42.

[27] Bogaerds, A. C. B. , Hulsen, M. A. , Peters, G. W. M. , Baaijens, F. P. T. , J. Rheol. 2004, 48, 765–785.

[28] Brooks, A. N. , Hughes, T. J. R. , Comp. Meth. Appl. Mech. Eng. 1982, 32, 199–259.

[29] Hulsen, M. A. , Fattal, R. , Kupferman, R. , J. Non‐Newtonian Fluid Mech. 2005, 127, 27–39.

[30] D'Avino, G. , Maffettone, P. L. , Greco, F. , Hulsen, M. A. , J. Non‐Newtonian Fluid Mech. 2010, 165, 466–474.

[31] Hu, H. H. , Patankar, N. A. , Zhu, M. Y. , J. Comp. Phys. 2001, 169, 427–462.

